# Perforated jejunal diverticulitis: a rare but important differential in the acute abdomen

**DOI:** 10.1186/s40792-020-00929-3

**Published:** 2020-07-06

**Authors:** Natasha Leigh, Brianne J. Sullivan, Roi Anteby, Susan Talbert

**Affiliations:** 1 Department of General Surgery, Icahn School of Medicine at Mount Sinai St. Luke’s Roosevelt Hospital, 425 West 59th Street, Suite 7B, New York, NY 10019 USA; 2 Faculty of Medicine, Tel Aviv University, Tel Aviv, Israel

**Keywords:** Jejunal diverticulitis, Abdominal pain, Acute abdomen, Perforation, Diverticulitis

## Abstract

**Background:**

Diverticulosis of the small bowel is rare and, in most cases, discovered incidentally. However, diverticulitis and other complications are important to consider in the differential of an acute abdomen, especially in the elderly population.

**Case presentation:**

The patient was a 59-year-old female who presented with acute lower abdominal pain progressing to peritonitis. Computed tomography scan showed a large inflamed and perforated diverticulum on the mesenteric side of the jejunum. Exploratory laparotomy revealed a dilated proximal jejunum with a 5-cm inflamed and perforated mesenteric diverticulum. A small bowel resection with primary anastomosis was performed.

**Conclusions:**

Jejunal diverticulitis remains a diagnostic challenge. Although uncommon, owing to its high mortality rate, it is an important clinical entity to consider and requires timely management.

## Background

Jejunal diverticulosis is a relatively rare condition with a reported annual incidence of 0.3–2.3% [1]. The majority of cases are asymptomatic and found incidentally either on computed tomography (CT) scan or at the time of operation for an unassociated condition. In a percentage of patients, akin to colonic diverticulosis, they can become acutely inflamed (diverticulitis) or have a more complicated presentation including perforation, intestinal bleeding, or obstruction [2]. Owing to the rarity of this condition and varied presentation, clinical diagnosis alone remains challenging and adjunctive imaging techniques are commonly required in order to form a prompt diagnosis.

## Case presentation

A 59-year-old female presented to the emergency department with 48 h of sudden onset abdominal pain. The pain began across the lower abdomen and subsequently became diffuse involving all four quadrants. Associated symptoms included nausea without vomitus and fever. Her past medical history was significant for hypertension, type II diabetes mellitus, depression, and one episode of colonic diverticulitis managed with antibiotics alone. Vital signs were abnormal with a fever of 102 °F and tachycardia of 121 bpm, but normotensive 129/75 mmHg. On physical examination, the patient was tender to palpation throughout the lower and mid abdomen without rebound or guarding and had some fullness over the umbilical region.

### Diagnostic studies

Laboratory results were notable for an elevated white blood cell count (15.3) with 81% neutrophilia. All other labs were within normal limits including blood urea nitrogen (BUN) (13), creatinine (0.92), and lactic acid (1.27).

**Fig. 1:**
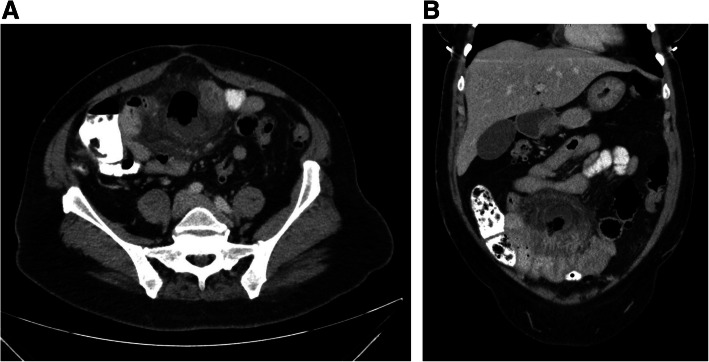
CT of the abdomen with oral and IV contrast. **a** Axial image of the mid-jejunal diverticulum with perforation. **b** Coronal image demonstrating associated edema of the jejunal loop and adjacent mesentery

A CT scan of the abdomen with oral and intravenous (IV) contrast revealed a jejunal loop with a large diverticulum on the mesenteric side with associated diverticulitis and a 5.3 × 3.6 × 4.8 cm contained perforation. There was extensive edema of the entire jejunal loop and hazy infiltration of the adjacent mesentery (Fig. 1a, b). No arrowhead sign (an arrowhead-shaped collection of extraluminal air packed between the perforated diverticulum and inflamed mesentery) was seen in this case. There was also no lymphadenopathy or ascites seen. There were also multiple duodenal, jejunal, ileal, and colonic diverticula without evidence of diverticulitis.

The patient was admitted with the diagnosis of jejunal diverticulitis with perforation but without clinical peritonitis. She was taken emergently to the operating room and underwent an exploratory laparotomy via midline incision. On entering the abdomen, a small amount of serous fluid was encountered without frank purulence. The involved jejunal loop was found to have serosal purulent exudates as well as interloop adhesions. The proximal jejunum was dilated and the distal jejunum collapsed. Twenty-seven centimeters of the proximal jejunum was resected to healthy non-inflamed edges, and a stapled side-to-side functional end-to-end anastomosis was performed. The small bowel was run from the ligament of Treitz to the ileocecal junction, and 4 other large non-inflamed diverticula ranging from 0.5 to 3 cm in diameter were seen along the mesenteric border. These were widely spaced and therefore not excised, as this would have required multiple further small bowel resections and anastomoses with associated increased morbidity. The abdomen was lavaged and closed.

### Pathology

**Fig. 2:**
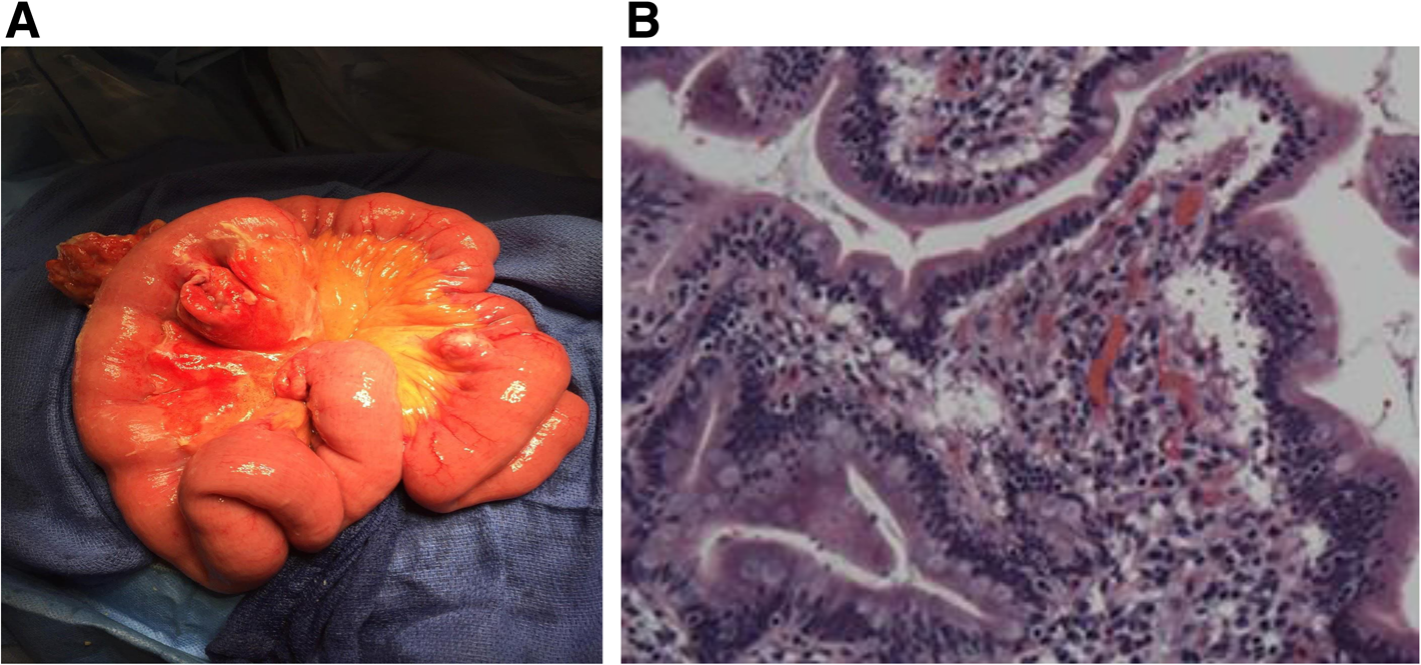
Pathology. **a** Gross specimen of the resected jejunal segment containing the perforated diverticulum. **b** Histologic examination with transmural jejunal inflammation, serosal inflammatory exudate, and perforation

Gross examination of the specimen revealed a 27-cm length of the jejunum with focal areas of fibrinous material. Within the specimen were three diverticula originating from the mesenteric side of the jejunal serosa. On microscopic examination, one diverticulum demonstrated transmural inflammation with acute inflammatory exudate and perforation. The other two appeared non-inflammatory. The surrounding omentum was thickened with fibrinous exudates and focal areas of hemorrhage (Fig. 2a, b).

### Postoperative course

Postoperatively, the patient’s course was uncomplicated. She had return of bowel function, tolerated a regular diet, and was discharged on postoperative day 6.

## Discussion

Diverticular disease is a relatively common disorder and may be multifocal throughout the intestinal tract. Most frequently, it affects the colon; however, other sites include the jejunum, ileum, and duodenum. Jejuno-ileal diverticulosis was first described in 1794 by Somerling. It is relatively rare, comprising only 18% of all small bowel diverticula, which is likely an underestimate given they are often incidentally found on imaging [3–5]. Their etiology is unclear; however, some studies have postulated that abnormal neuromotor innervation causing intestinal dyskinesia may be a factor [6]. This causes abnormally elevated intraluminal pressure resulting in the formation of false (pulsion) diverticula at points of weakness along the intestine where blood vessels penetrate the wall, similar to colonic diverticulosis. Unlike their true counterparts (to include Meckel’s diverticula), they involve only the mucosal and submucosal layers of the bowel wall and are typically located on the mesenteric border [5, 7]. There appears to be an association with age older than 60 years, male gender, colonic diverticulosis, and systemic connective tissue diseases [2, 6]. Familial tendencies have also been noted.

Table 1 demonstrates cases of jejunal diverticulosis with diagnosis and management reported in the literature over the last 10 years. Comparing this case to others reported in literature, our patient was younger in age (mean age = 74). She presented with an acute abdomen, whereas most small bowel diverticula are asymptomatic. The diverticula were found on the mesenteric side of the bowel, the most common location for jejunal diverticulosis. In cases published in the last decade, only one report stated finding anti-mesenteric small bowel diverticula [40]. Our patient was treated with an open surgical resection, as reported in the majority of the cases. Of note, there were reports of conservative treatment and laparoscopic resection [5, 7, 9, 12, 14, 17, 19, 25, 28, 31], but not in patients with perforated diverticulitis presenting with an acute abdomen.

**Table 1:** Characteristics and treatment of jejunal diverticulosis in cases published between 2010 and 2020

Paper	Year	Age	Gender	Preoperative diagnosis	Distance from Trietz’s lig. (cm)	Location	No. of diverticula	No. of perforations	Type	Diverticulitis	Treatment
Prough H [8]	2019	65	M	Diverticulitis	NA	Mesentery	1	0	NA	Yes	Open surgery with resection
Gurala D [9]	2019	70	F	Small intestine diverticulitis	NA	NA	2	1	NA	Yes	Failed conservative, laparoscopic surgery with resection
Mazahreh TS [10]	2019	68	M	Gastrointestinal bleed	10	NA	Multiple	0	True and false	No	Open surgery with resection
Fleres F [11]	2018	88	F	Unknown	NA	NA	3	1	NA	Yes	Open surgery with resection
Fleres F [11]	2018	86	F	Volvulus	150	Mesentery	Multiple	0	NA	Yes	Open surgery with resection
Abdelbaki A [12]	2018	65	F	Small intestine diverticulitis	45	NA	Multiple	1	NA	Yes	Open surgery with resection
Abdelbaki A [12]	2018	74	F	Small intestine diverticulitis	NA	NA	NA	0	NA	Yes	Conservative
Abdelbaki A [12]	2018	87	M	Small intestine diverticulitis	NA	NA	NA	0	NA	Yes	Conservative
Syllaios A [13]	2018	75	M	Small intestine diverticulitis	NA	NA	6	0	False	Yes	Open surgery with resection
Kagolanu DC [5]	2018	91	M	Small intestine diverticulitis	NA	NA	Multiple	0	NA	Yes	Conservative
Ejaz S [14]	2017	76	M	Small intestine diverticulitis	NA	NA	Multiple	NA	NA	Yes	Conservative
Ejaz S [14]	2017	78	F	Small intestine diverticulitis	NA	NA	Multiple	0	NA	Yes	Conservative
Ejaz S [14]	2017	87	M	Small intestine diverticulitis	NA	Mesentery	Multiple	NA	NA	Yes	Conservative
Grubbs J [15]	2017	90	M	Sigmoid diverticulitis	NA	NA	Multiple	1	NA	Yes	Open surgery with resection
Kumar D [16]	2017	60	F	Small intestine diverticulitis	NA	Mesentery	Multiple	0	NA	Yes	Open surgery with resection
Kumar D [16]	2017	68	M	Small intestine diverticulitis	NA	Mesentery	Multiple	1	NA	Yes	Open surgery with resection
Cui J [17]	2017	65	F	Small intestine diverticulitis	NA	Mesentery	Multiple	0	NA	Yes	Failed conservative, laparoscopic surgery with resection
Malghan L [18]	2017	91	F	Small intestine diverticulitis	NA	NA	Multiple	0	NA	Yes	Open surgery with resection
Karas L [19]	2017	82	F	Intestinal mass	NA	NA	Multiple	0	NA	Yes	Open surgery with resection
Karas L [19]	2017	80	F	Small intestine diverticulosis	NA	NA	Multiple	0	NA	Yes	Laparoscopic surgery with resection
Mohi RS [20]	2016	62	M	Volvulus	NA	Mesentery	Multiple	0	NA	Yes	Open surgery with resection
Aydin E [21]	2016	69	M	Small intestine diverticulitis	20	Mesentery	Multiple	0	NA	Yes	Open surgery with resection
Tenreiro N [22]	2016	81	M	Diverticulitis	NA	NA	Multiple	1	NA	Yes	Failed conservative, open surgery with resection
Ghrissi R [23]	2016	72	M	Small bowel obstruction	NA	NA	Multiple	0	NA	No	Open surgery with resection
Harbi H [24]	2016	31	M	Unknown	NA	NA	Multiple	1	False	Yes	Open surgery with resection
De Minicis S [25]	2015	60	M	Jejunal diverticula	NA	NA	Multiple	0	NA	Yes	Conservative
Natarajan K [26]	2015	56	M	Small intestine diverticulitis	8	Mesentery	Multiple	3	False	Yes	Open surgery with resection
Kassir R [27]	2015	79	M	Small intestine diverticulitis	NA	Mesentery	Multiple	1	NA	Yes	Open surgery with resection
Fidan N [7]	2015	67	M	Small intestine diverticulitis	NA	NA	Multiple	0	NA	Yes	Conservative
Levack MM [28]	2014	77	M	Small intestine diverticulum	NA	NA	1	1	NA	No	Conservative
Xu XQ [29]	2014	86	M	Small intestine diverticulitis	50	NA	Multiple	NA	NA	Yes	Open surgery with resection
Fresow R [30]	2014	73	M	Small intestine diverticulitis	0	NA	3	0	NA	Yes	Open surgery, no resection
Corcelles R [31]	2014	63	F	Intestinal perforation	NA	NA	Multiple	NA	NA	Yes	Laparoscopic resection
Ojili V [32]	2014	75	M	Small intestine diverticulitis	NA	NA	NA	NA	NA	Yes	Open surgery with resection
Zamani A [33]	2013	63	F	Small intestine diverticulitis	12	NA	Multiple	1	NA	Yes	Open surgery with resection
Aydin I [34]	2013	74	F	Small intestine diverticulitis	40–100	Mesentery	Multiple	1	False	Yes	Open surgery with resection
Singal R [35]	2012	63	M	Unknown	NA	NA	Multiple	0	NA	No	Open surgery, no resection
Ferreira-Aparicio FE [36]	2012	65	F	Appendicitis	0	NA	Multiple	Multiple	NA	Yes	Open surgery with resection and ileostomy
Ferrarese A [37]	2012	92	F	Intestinal perforation	NA	NA	NA	1	NA	Yes	Open surgery with resection
Garnet DJ [38]	2011	80	M	Small intestine diverticulitis	NA	NA	Multiple	1	NA	Yes	Laparoscopic converted open surgery, with resection
Tan KK [39]	2011	88	M	Gastrointestinal hemorrhage	NA	NA	NA	0	NA	No	Open surgery with resection
Tan KK [39]	2011	72	M	Gastrointestinal hemorrhage	NA	NA	NA	0	NA	No	Open surgery with resection
Tan KK [39]	2011	84	M	Gastrointestinal hemorrhage	NA	NA	Multiple	0	NA	No	Open surgery with resection
Tan KK [39]	2011	70	M	Intestinal inflammation	NA	NA	Multiple	1	NA	Yes	Open surgery with resection
Tan KK [39]	2011	84	M	Intestinal inflammation	NA	NA	1	1	NA	Yes	Open surgery with resection
Tan KK [39]	2011	75	M	Intestinal inflammation	NA	NA	Multiple	1	NA	Yes	Open surgery with resection
Nonose R [40]	2011	86	F	Intestinal perforation	15–50	Anti-mesenteric	Multiple	1	NA	Yes	Open surgery with resection
Falidas E [41]	2011	55	M	Small intestine diverticulum and bowel obstruction	NA	NA	Multiple	0	NA	Yes	Failed conservative, open surgery with resection
Sakpal SV [42]	2010	25	F	Enteritis	NA	Mesentery	1	1	NA	Yes	Open surgery with resection
França M [43]	2010	75	M	Small intestine diverticulitis	NA	Mesentery	3	1	NA	Yes	Open surgery with resection
Vanrykel F [44]	2010	79	F	Small intestine diverticulitis	NA	NA	Multiple	1	NA	Yes	Laparoscopic converted open surgery, with resection
Chugay P [45]	2010	89	F	Small intestine diverticula	NA	NA	Multiple	NA	NA	No	Failed conservative, open surgery with resection
Chugay P [45]	2010	79	M	Small intestine perforation	NA	NA	Multiple	1	NA	Yes	Open surgery with resection

PubMed database was queried for studies published from January 1, 2010 to April 31, 2020, with English language restriction. Search strategy included the term “jejunal diverticulitis.” Case series lacking patient-specific data were excluded

*Lig* ligament, *No.* number, *NA* not available, *F* female, *M* male

In the majority of cases, jejunal diverticulosis is diagnosed incidentally either on imaging or intraoperatively. However, around 10–30% of patients present with disease complications including diverticulitis, perforation, bleeding, or small bowel obstruction [46]. Isolated perforation is extremely rare, and to date, only a few cases have been reported in the English literature. Consequently, most centers have little experience with managing these cases. It is believed that in the same manner that asymptomatic colonic diverticulosis is managed without intervention, incidental jejunal diverticulosis is not of clinical significance. However, the diagnosis and management of its complications remains a challenge. Patients typically present with non-specific symptomatology including acute abdominal pain in varied locations, fever, intestinal bleeding, and obstructive symptoms meaning that there is reliance on adjunctive tools such as CT scans or balloon enteroscopy and often a delay in making the correct diagnosis [47]. Many have adopted management strategies with a similar approach to colonic disease. Non-operative treatment with bowel rest and antibiotics has been successful for cases of uncomplicated diverticulitis [5]. However, in patients who present with acute peritonitis, hemodynamic instability, or evidence of free perforation, an aggressive operative approach is most appropriate. On review of complicated diverticulitis cases to date, most authors have had successful outcomes with resection of the involved small bowel segment and primary anastomosis [4, 46, 48]. This is especially important when considering the high mortality (around 20–30%) associated with this disease process, mostly attributable to a delay in diagnosis. Our patient was managed in this manner and successfully discharged without the development of complications.

## Conclusion

Complicated jejunal diverticulitis can be both a diagnostic and therapeutic challenge with a high mortality rate. We recommend that jejunal diverticulitis be considered a differential diagnosis in the acute abdomen and a CT scan will allow for the timeliest diagnosis. In cases of free perforation, operative resection of the affected segment with primary anastomosis appears to be a successful management strategy.

## Data Availability

This is a case report, so there is no dataset. The data for the patient, however, is available upon request.

## Abbreviations

CT: Computed tomography
BUN: Blood urea nitrogen
IV: Intravenous
